# The role of family-school relationship in promoting preschoolers’ classroom engagement

**DOI:** 10.3389/fpsyg.2025.1615150

**Published:** 2025-08-05

**Authors:** Luyou Shen

**Affiliations:** Shanghai Institute of Early Childhood Education, Shanghai Normal University, Shanghai, China

**Keywords:** student engagement, behavioral engagement, cognitive engagement, emotional engagement, family-school relationship, preschool children

## Abstract

In 1993, Finn proposed in his paper that classroom engagement can be described from three dimensions. Subsequently, research on children’s classroom engagement gradually gained attention. Although previous literature has explored the factors that affect classroom participation, most studies only focus on the influence of family or school, rarely considering the family-school relationship as a whole. This study aims to address this deficiency by systematically reviewing literature and using the PRISMA search strategy to exclude non-English literature, books, and conference papers. I have also developed three screening criteria: (1) research topic is not on home-school relationship; (2) research topic is not on students’ participation; (3) research participants are not preschool children. Finally, 17 relevant articles were selected for analysis. Research has founfd that good family-school relationship can enhance children’s classroom participation, manifested in parents accompanying their children more in learning, helping them improve social skills and develop good habits. Parents and teachers will trust each other, reduce information gaps, and enhance children’s psychological security. And they will cooperate to provide opportunities for children to exercise their cognitive abilities and enhance their cognitive participation. This study provides valuable reference for educators in the field of preschool education to understand the relationship between family-school relationship and children’s classroom participation. Therefore, educators are able to adjust their educational strategies and design courses that can fully mobilize children’s enthusiasm and help them grow.

## 1 Introduction

As the starting point of children’s education, the preschool education stage significantly influences their future learning and lifelong development. During this critical period, the performance of children in the classroom has become a focus of attention for both teachers and parents. Class engagement is an important indicator for measuring children’s learning engagement and educational effectiveness, which is directly related to their cognitive development, social skills, and emotional health (McConnell and Kubina Jr, 2014; Serpell and Mashburn, 2012; Smith et al., 2020). In the last two decades, researches on children’s class engagement have grown, and it’s now widely recognized that home-school relationship has been influential in affecting children’s engagement (Galindo and Sheldon, 2012; Riggs and Medina, 2005; Serpell and Mashburn, 2012). The home-school relationship, as a bridge connecting families and schools, plays a crucial role in enhancing children’s classroom participation. A good home-school relationship can promote communication and cooperation between families and schools, creating a more inclusive and motivating learning environment. This environment allows children to fully unleash their potential, actively participate in the classroom, and lay a solid foundation for their personal growth.

Through reading literature, I found that researchers have extensively discussed the relationship between family-school relationship and children’s engagement. For example, Serpell and Mashburn (2012) mentioned that the degree of closeness between home and school is positively correlated with children’s engagement. High-quality home-school communication can create opportunities for two-way information exchange, aligning the goals of parents and teachers, which helps to enhance students’ awareness of the school and increase their participation in it (Serpell and Mashburn, 2012). Malczyk and Lawson (2019) mentioned that parents have high expectations for their children’s academic trajectory, such as wanting them to achieve specific grades, which can be transmitted to them and motivate them to participate more actively in classroom learning.

I found that these literatures mostly study the impact of family or school on children’s classroom engagement separately (Fantuzzo et al., 2013; Froiland, 2021; Malczyk and Lawson, 2019), and rarely connect the two to study the influence of family-school relationships on children’s classroom engagement (Serpell and Mashburn, 2012; Smith et al., 2020). The relationship between family and school can have an impact on various aspects such as parental guidance for children’s learning, thereby affecting children’s class participation.

Given the theoretical positions taken for the study and the status of the field as briefly reviewed above, the study aimed to provide an answer to the following questions:

1. What is students’ engagement?
2. What is the impact of home-school relationship on children’s engagement?

Through this study, I aim to identify and reinforce key elements that can promote active participation of young children in classroom activities, thereby enhancing the overall quality of early childhood education. By reading this article, early childhood educators can adjust their educational strategies, optimize communication and cooperation with parents. Parents can recognize their role and influence in early childhood education and learn how to participate more effectively in their children’s learning process.

The rest of the article includes method、results and conclusion. The method section will introduce the keywords used in this article and the methods used for data collection. The results section will provide specific answers to the two main questions raised in the previous text. The conclusion section will summarize the research content of this article, introduce the limitations and advantages of the article, and discuss how future research should be conducted.

## 2 Methods

### 2.1 Search strategy

The process of article selection followed the Preferred Reporting of Items for Systematic Reviews and Meta-Analyses (PRISMA) Statement. I searched Scopus and Web of Science on November 30th, 2024 for peer-reviewed articles on home-school relationship and class participation. I operationalized different permutations of each keyword based on previously validated searches. For home-school relationship, I included “family-school,” “family-school relationship,” following Semke and Sheridan’s review (2012) which is published in the *School Community Journal* and Smith et al.’s review (2020) which is published in the *Educational Psychology Review*. For class engagement, I drew on Quin’s review (2017) and Martins et al.’s review (2022) to identify keyword variants. In short, by using search terms that have been validated in publications from prestigious journals, this made me more confident that I would capture appropriate citations within the searches that we did conduct.

I applied the fields title/abstract in the search. The full details are available in Appendix 1. My initial search identified a total of 249 articles in Web of’ Science and 326 in Scopus, which were imported into Zotero reference management software. 0f these 575 articles, 155 were identified as duplicates, leaving a total of 420 for screening and eligibility stages.

### 2.2 Inclusion and exclusion

**Figure 1:**
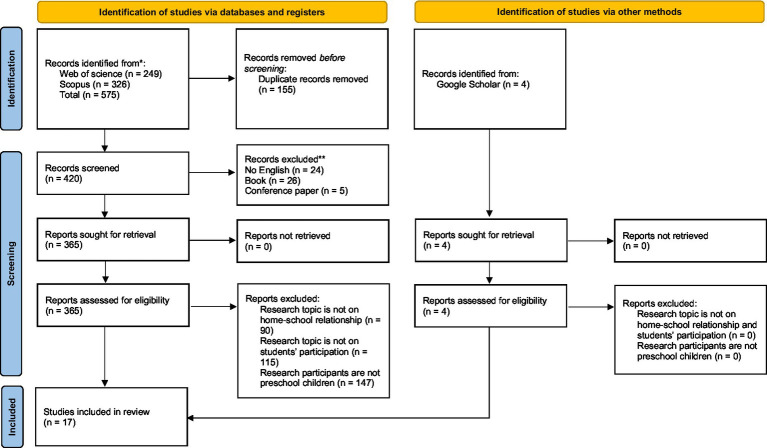
PRISMA 2020 flow diagram for new systematic reviews which included searches of databases, registers and other sources. *Consider, if feasible to do so, reporting the number of records identified from each database or register searched (rather than the total number across all databases/registers). **If automation tools were used, indicate how many records were excluded by a human and how many were excluded by automation tools. Source: Page MJ, et al. BMJ 2021; 372:n71. doi: 10.1136/bmj.n71.

I applied a series of inclusion and exclusion criteria. Articles were included if they were: (i) written in English; (ii) published in a peer-reviewed journal. They are excluded if the research design is book, Conference paper, etc. This is because these research types are not relevant to our research questions. Of the 420 records screened, I excluded 31 because they were book chapters and conference papers, and 24 were not in English, leaving a total of 365 articles for retrieval. I was able to find the full text of all articles, resulting in 365 articles for eligibility, At eligibility, upon reviewing the full text, I excluded another 90 articles that did not relate to home-school relationship. I excluded another 115 articles that did not relate to class participation. I also further excluded 147 articles because the populations in these studies were not preschool children. I also identified additional 4 articles by scanning the reference list and then using Google Scholar. This left a final 17 articles in the final review sample for data analysis. Figure 1 further describes the process of inclusion/exclusion.

## 3 Results

### 3.1 What is students’ engagement?

Some papers indicate an intention to focus on class participation via their titles, keywords, or abstracts (Jeon et al., 2021; Pilarz et al., 2024; Smith et al., 2020). For example, school engagement is a multidimensional construct that includes behavioral, emotional, and cognitive components (Fredricks et al., 2004). Participation is the extent to which a youngster regularly participates in classroom and school activities (Finn and Voelkl, 1993). Regardless of whether these papers focused on multiple or single definitions, each paper included in the review reflected the following claims about the definition of students’ engagement:

1. Students’ engagement includes behavioral engagement, which refers to students’ participation in learning activities, including attention, positive behavior, and attendance rate.
2. Students’ engagement includes emotional engagement, which refers to students’ emotional attitudes, sense of identity, and sense of belonging to the classroom.
3. Students’ engagement includes cognitive engagement, which refers to the use of self-regulated learning methods and metacognitive strategies, utilizing shallow and deep learning strategies to learn and understand materials.

We illustrate each of these claims here by referencing a range of papers from different contexts and focusing on different interpretations.

With respect to the first knowledge claim, Finn and Voelkl (1993) discussed the meaning of students’ engagement from behavioral aspects. They noted that participation is the extent to which a youngster regularly participates in classroom and school activities, involving attending class, paying attention to the teacher, and responding to questions (Finn and Voelkl, 1993). This article makes the abstract concept of “student participation” concrete. This clear definition is helpful for researchers to conduct empirical research and also facilitates teachers to monitor and record students’ participation in daily teaching.

With respect to the second knowledge claim, Li and Lerner (2013) discussed the meaning of students’ engagement from emotional aspects. They noted that active participation, with great concentration and effort, positive emotions, or feeling of excitement and sense of connectedness is a necessary ingredient for students’ engagement with school (Li and Lerner, 2013). This viewpoint breaks through the limitations of defining student participation solely from a behavioral perspective. This multidimensional definition more comprehensively reflects the essence of student participation, as students’ learning in school are complex processes that are influenced by multiple factors. And high students’ emotional engagement means that they do not just participate passively but actively engage in learning based on their beliefs and values.

With respect to the third knowledge claim, Naibert and Barbera (2022) discussed the meaning of students’ engagement from cognitive aspects. They noted that cognitive engagement is often considered in relation to students’ psychological investment in their learning, which includes putting in effort to understand and master the material (Naibert and Barbera, 2022). This viewpoint links cognitive engagement with students’ diligent mastery of learning materials, which is in line with the essential characteristics of student learning. And it is pointed out that students should actively explore deeper knowledge and expand their thinking horizons, which helps them cultivate their innovation ability and higher-order thinking.

Overall, classroom participation refers to the degree to which students regularly participate in the classroom. Students appear in the classroom and are able to express their thoughts. If the teacher initiates a discussion, raises questions or difficulties in class, students actively discuss and answer, rather than just sitting in the classroom. At the same time, students have a sense of identity and belonging to the classroom, enjoy the class, and are able to understand the teacher’s words seriously in class.

### 3.2 What is the impact of home-school relationship on children’s engagement?

A good home-school relationship can enhance children’s class engagement. Firstly, in a good home-school relationship, parents will pay more attention to accompanying their children’s learning. These companions can help young children improve their social skills (Jeon et al., 2021; Serpell and Mashburn, 2012), develop good habits such as sitting still and concentration (Galindo and Sheldon, 2012; Son and Tineo, 2016), and thus help them better adapt to the class and enhance their behavioral participation. For example, Johnson et al. (2012) presented a guessing game in his article. Children are asked to feel for an object in a paper bag and guess the identity of the object. He noted that:

In this activity, children use expressive language to describe the object, parents provide additional descriptions, and finally the object is revealed to the children. Parents interact with their children to develop their physical skills by writing, drawing, coloring, and moving their bodies in games and activities (Johnson et al., 2012, p. 5).

In my opinion, this example demonstrates how parental involvement in such home-based learning activities can have a positive impact on children. When parents actively participate in their children’s learning process, they can guide children to use more accurate and vivid words to describe the object, which enriches children’s vocabulary. And they can guide children to draw to improve children’s fine motor skills. At the same time, this kind of interaction between parents and children can help children improve their social skills. I believe that if children are accustomed to an interactive and engaging learning environment at home, they are more likely to expect similar experiences in the class. For instance, they are more willing to answer questions and participate in discussions in class because they have developed confidence and communication skills through family activities.

Secondly, parents and teachers will trust each other and jointly support children’s learning. Emotional factors such as mutual trust and support play a key role in the impact of home-school relationship on children’s participation (Serpell and Mashburn, 2012). Premo also noted that parents affirmed the importance of teachers being honest about challenges their child is facing and helping parents overcome them. Many parents will seize the time after school to communicate with teachers about their children’s study (Premo et al., 2023).

From my internship experience, I also found that the pick-up and drop off time are a critical time for parents to contact and ask questions from their children’s teachers. Parents who trust their teachers will use this opportunity to discuss their children’s learning situation. This behavior may break the information gap between family and school. Teachers can develop learning strategies that are more suitable for the current situation of children. Parents can assist teachers in teaching activities by exercising their children’s expression skills, etc. In this situation, children can feel the support and attention from both their family and school. This kind of support may make children feel more psychologically secure in the classroom. Moreover, by communicating with parents to understand their children’s hobbies, teachers can design classes that are in line with children’s interests, making children more willing to participate in the classroom.

Thirdly, parents and teachers will work together to provide opportunities for children to exercise their cognitive abilities, such as helping them learn to think. When I was on my internship, I found that the teacher taught children how to understand the meaning conveyed by the story when children were reading stories. After class, she let parents continue the classroom learning content at home. For example, they can read the picture books mentioned in class with their children and discuss the content of stories, so as to strengthen the children’s learning experience in the classroom.

This example demonstrates that children received good support for learning and thinking. In kindergarten, teachers actively guide children to master the correct learning methods, such as helping children understand the logic of stories. Parents cooperate with teachers at home by practicing with their children themselves, such as reading picture books together. As Son and Tineo (2016) said, parent–child shared reading help children learn to think about story content on their own, and this ability will be directly applied in the classroom. At first, children may just listen to the plot of stories. With the guidance of teachers and parents, they can gradually understand the meaning behind stories and eventually tell stories to others in their own words. During this process, children’s thinking ability continue to improve, and they will be more willing to think actively in class.

## 4 Conclusion and limitation

This study took qualitative research, focusing on how a good home-school relationship improve children’s classroom engagement. Following the steps of the evaluation logic, we developed 6 evaluation standards. We believe that these standards cover the key elements of qualitative research quality, including the clarity of the statement of research purpose, the quality of reference citations, and the overall credibility of the research. This study’s results indicate that, the level of children’s classroom engagement can be measured from three aspects: behavioral engagement, emotional engagement, and cognitive engagement. In a good home-school relationship, parents will be more deeply involved in their children’s learning, helping children develop their social interaction skills. And they will actively communicate with teachers about children’s problems that occur in the classroom. Both parents and teachers will jointly develop targeted educational strategies to provide more comprehensive support and guidance for children, facilitating the balanced development of children in different aspects such as behavior and cognition, and comprehensively enhance their classroom participation.

At present, we acknowledge that the study only used two databases (Web of science, Scopus), which may lead us to overlook some literature from other databases, such as EBSCO and CNKI. We may miss the relationship between schools and parents from different countries and cultures, and whether it will have different impacts on children’s classroom engagement. Despite these limitations, we still followed the systematic data collection strategy, reported keywords openly and transparently, and built our keywords on previous research. By following the PRISMA search strategy, we systematically excluded non-English language articles、book and conference paper. Through these methods, I’m able to search for articles related to my research. In addition, adhering to the principle of honesty, we extracted and summarized all the included articles into an Excel spreadsheet, and conducted a detailed review of each article. In summary, the evaluation data are quite reliable for readers.

Future research can be further expanded from multiple perspectives. In terms of research methods, we can try to use new technological means such as eye tracking, EEG monitoring, etc. to accurately measure children’s classroom participation and comprehensively analyze it. In the field of practical application, it is possible to develop a home-school cooperation training program to provide operational methods and practical cases for schools and parents. Intervention research can also be conducted to select classes or schools with poor home school relationships to implement intervention measures based on research results and verify their effectiveness in practice. Through these multidimensional in-depth studies, it is expected to more comprehensively and accurately reveal the impact mechanism of home school relationships on children’s classroom participation, thereby providing more solid theoretical and practical support for optimizing home school cooperation and improving the quality of early childhood education.
