# Supplementary material for: The role of family-school relationship in promoting preschoolers’ classroom engagement
Source: Front Psychol. 2025 Aug 5;16:1615150. doi: 10.3389/fpsyg.2025.1615150 (PMC12392103; doi:10.3389/fpsyg.2025.1615150)
Supplement: Supplementary file 1 [file Supplementary_file_1.pdf]

Appendix 1.

Table 1.

Terms used to search two databases related to qualitative research on family-school relationship and classroom engagement.

Step	Terms	Results
Database: Web of science		
1	TS=("student engagement" OR "learner engagement" OR "academic engagement" OR "school engagement" OR "disengagement" OR "expulsion" OR "suspension" OR "dropout" OR "attendance" OR "truancy" OR "academic failure" OR "underachievement" OR "low achievement" OR "retention" OR "refusal" OR "behavioral engagement" OR "cognitive engagement" OR "emotional engagement")	873093
2	TS= ("family–school" OR "family–school partnership" OR "parent–school partnership" OR "family–school relationship" OR "parent–school relationship" OR "family involvement" OR "parent involvement")	6955
3	TS= ("toddlers" OR "preschool children" OR "children" OR "preschoolers" OR "preschool-aged children" OR "child")	2378078
4	1 AND 2 AND 3	249
Database: Scopus		
1	TITLE-ABS-KEY ("student engagement" OR "learner engagement" OR "academic engagement" OR "school engagement" OR "disengagement" OR "expulsion" OR "suspension" OR "dropout" OR "attendance" OR "truancy" OR "academic failure" OR "underachievement" OR "low achievement" OR "retention" OR "refusal" OR "behavioral engagement" OR "cognitive engagement" OR "emotional engagement")	749142
2	TITLE-ABS-KEY ("family–school" OR "family–school partnership" OR "parent–school partnership" OR "family–school relationship" OR "parent–school relationship" OR "family involvement" OR "parent involvement")	4243
3	TITLE-ABS-KEY ("toddlers" OR "preschool children" OR "children" OR "preschoolers" OR "preschool-aged children" OR "child")	8198659
4	1 AND 2 AND 3	326
